# Supplementary material for: The Use of a Three-Fluid Atomising Nozzle in the Production of Spray-Dried Theophylline/Salbutamol Sulphate Powders Intended for Pulmonary Delivery
Source: Pharmaceutics. 2020 Nov 20;12(11):1116. doi: 10.3390/pharmaceutics12111116 (PMC7699582; doi:10.3390/pharmaceutics12111116)
Supplement: Supplementary file 1 [file pharmaceutics-12-01116-s001.pdf]

pharmaceutics

Article

The Use of a Three-Fluid Atomising Nozzle in the Production of Spray-Dried Theophylline/Salbutamol Sulphate Powders Intended for Pulmonary Delivery

Stefano Focaroli [1,*], Guannan Jiang [1], Peter O'Connell [1], John V. Fahy [2] and Anne-Marie Healy [1,3]

1. School of Pharmacy and Pharmaceutical Sciences, Panoz Institute, Trinity College Dublin, Dublin 2, D02, Ireland; gjiang@tcd.ie (G.J.); peoconne@tcd.ie (P.O.); healyam@tcd.ie (A.M.H.)
2. Division of Pulmonary and Critical Care Medicine, Department of Medicine and Cardiovascular Research. Institute, Health Sciences East, UCSF, 513 Parnassus Avenue, San Francisco, CA, 94143, USA; john.fahy@ucsf.edu
3. SSPC The SFI Research Centre for Pharmaceuticals, School of Pharmacy and Pharmaceutical Sciences, Trinity College Dublin, Dublin 2, D02, Ireland

* Correspondence: focarols@tcd.ie; Tel.: +353-89-2414038

Supplementary materials

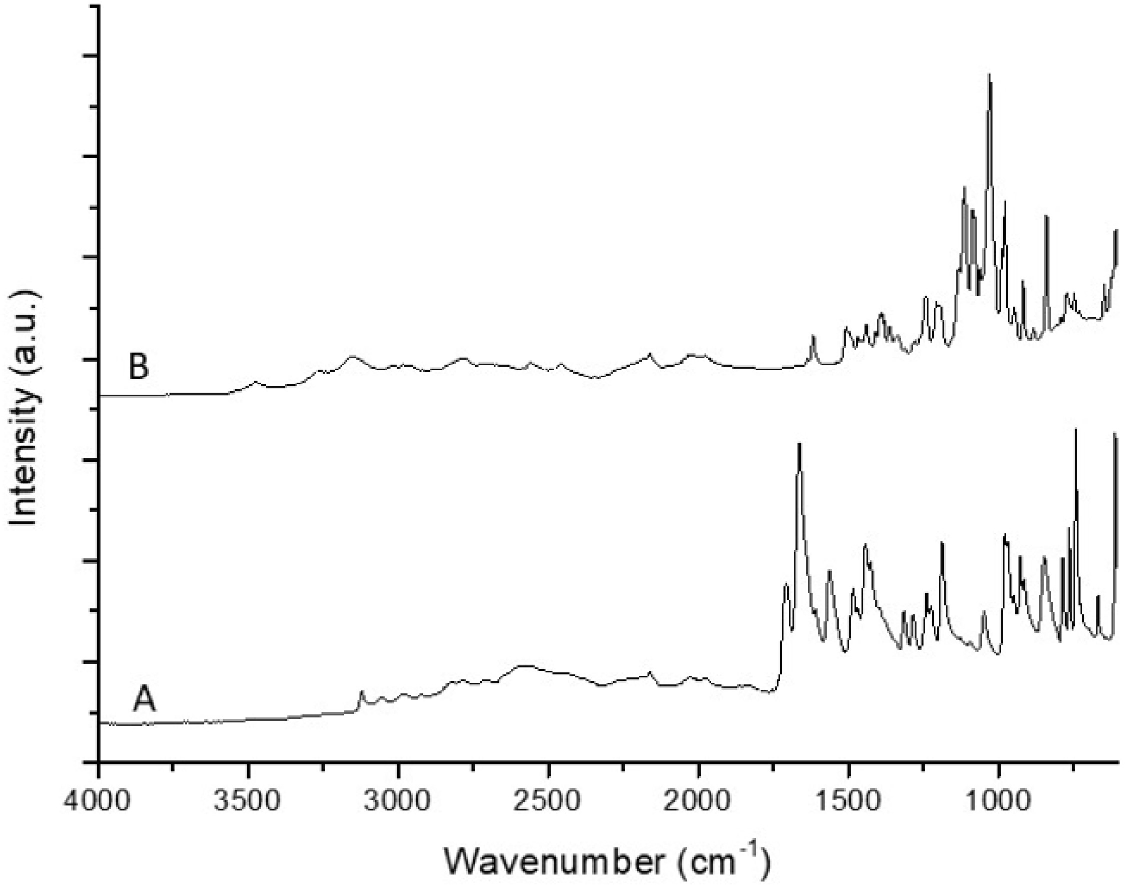

Figure S1. Infrared spectra of (A) theophylline and (B) salbutamol sulphate raw materials.

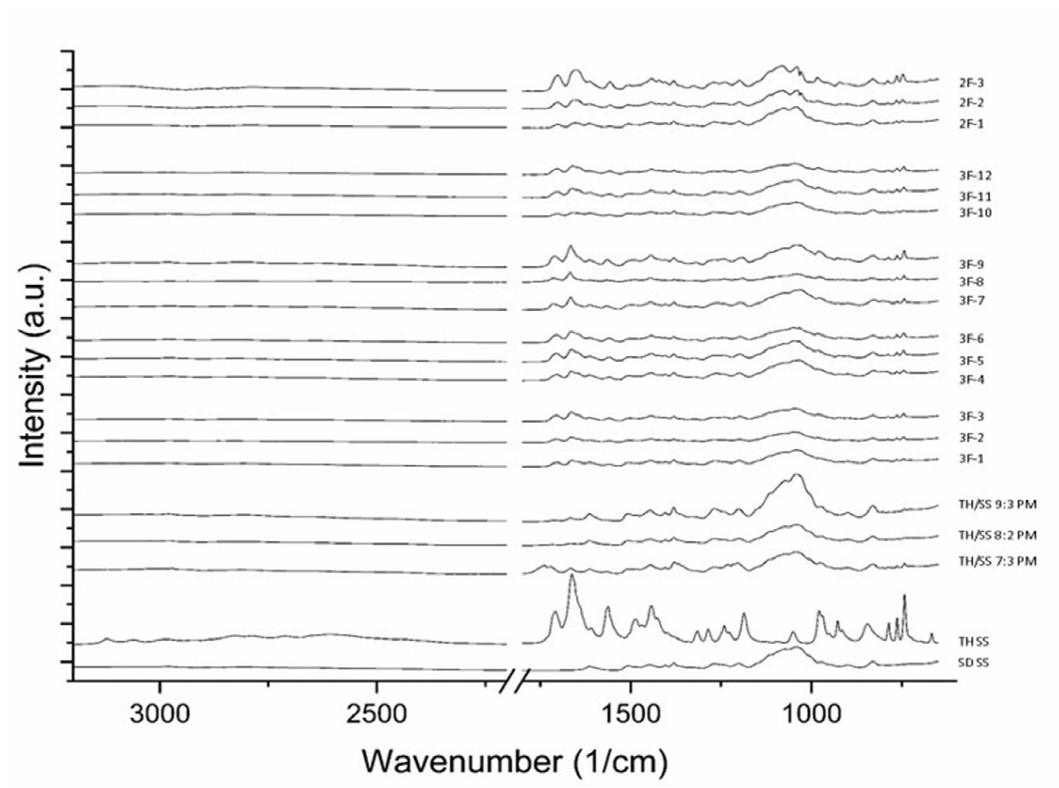

Figure S2. Infrared spectra of spray-dried formulations.

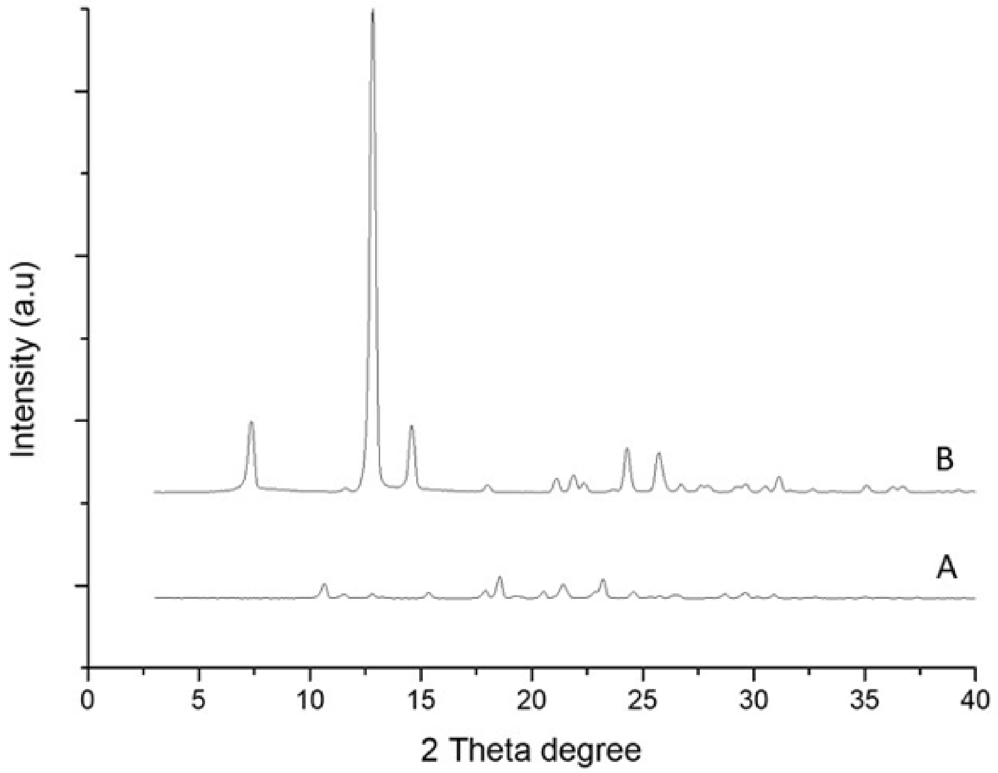

Figure S3. PXRD patterns of (A) salbutamol sulphate and (B) theophylline raw materials.

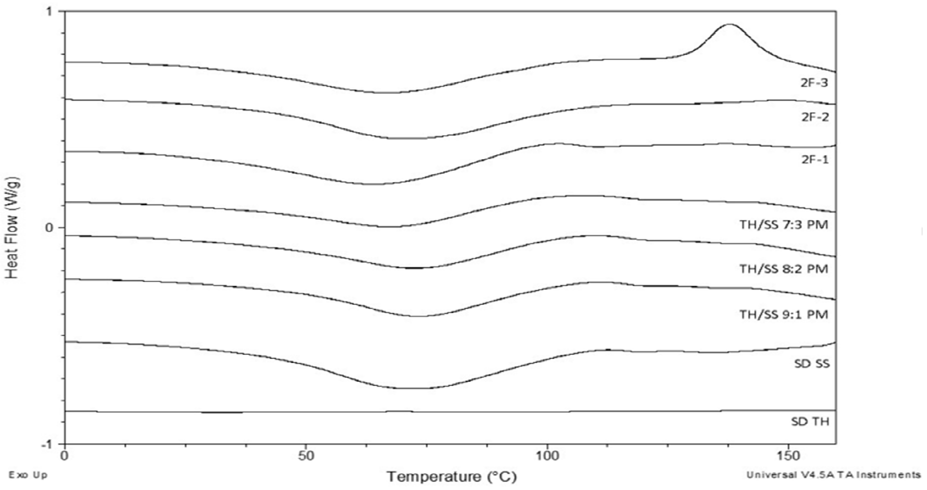

(A)

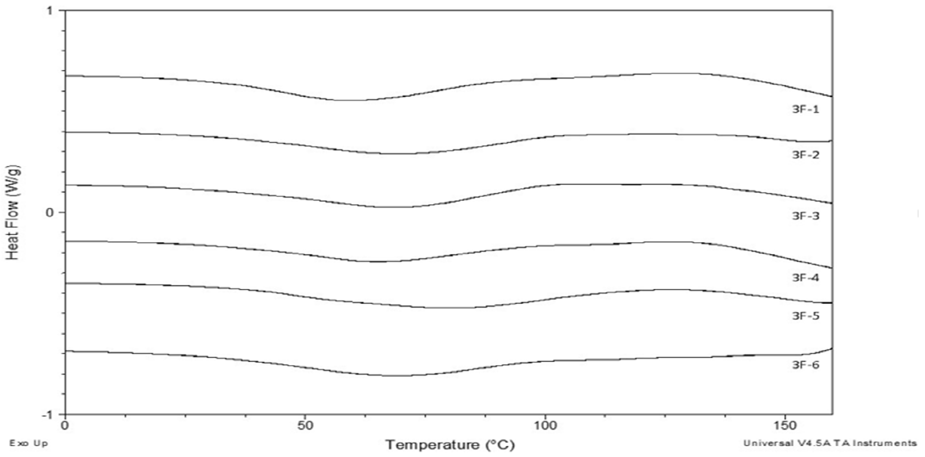

(B)

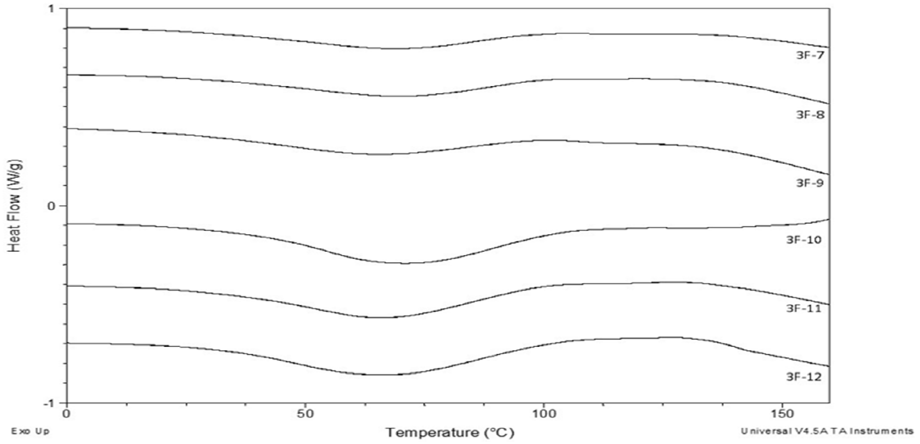

(C)

Figure S4. Representative DSC thermograms of powders, including (A) spray-dried theophylline; spray-dried salbutamol sulphate, spray-dried SS and TH physical mixture, samples 2F_1–3; (B) samples 3F_1–6; (C) samples 3F_7–12.

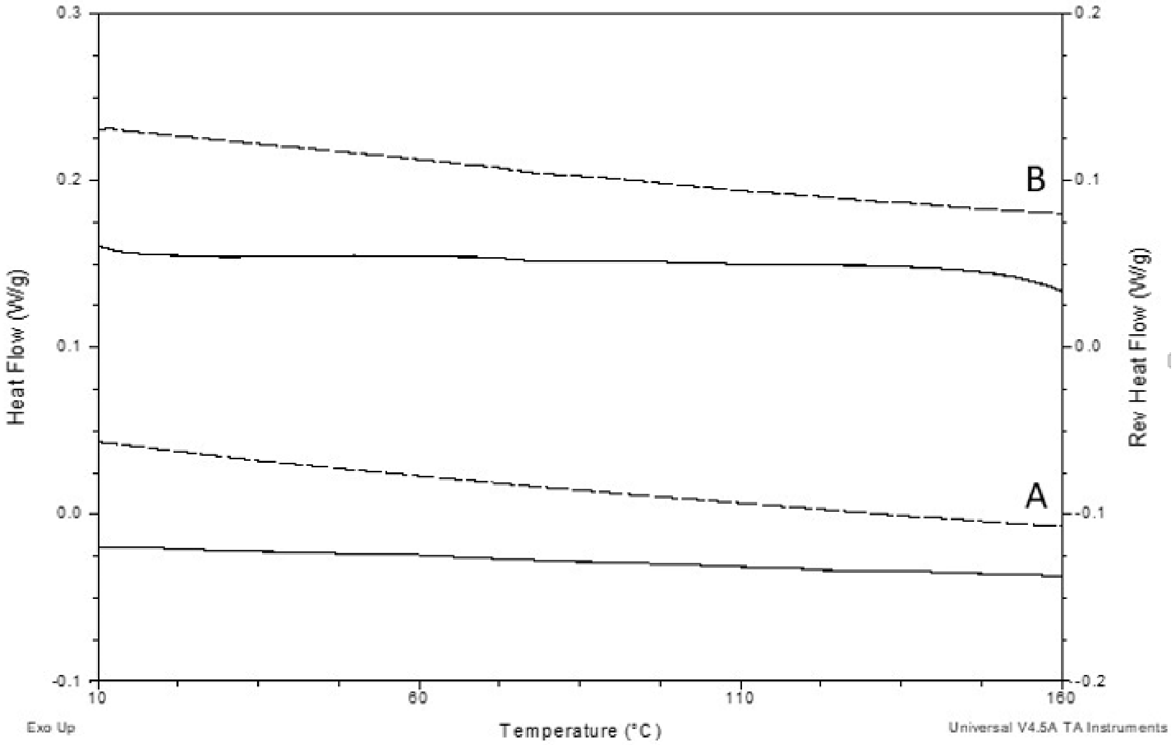

Figure S5. Reversible (dashed lines) and total heat flow (solid lines) MDSC thermograms of (A) spray-dried theophylline and (B) spray-dried salbutamol sulphate.
